# Satellite reveals a steep decline in China’s CO_2_ emissions in early 2022

**DOI:** 10.1126/sciadv.adg7429

**Published:** 2023-07-21

**Authors:** Hui Li, Bo Zheng, Philippe Ciais, K. Folkert Boersma, T. Christoph V. W. Riess, Randall V. Martin, Gregoire Broquet, Ronald van der A, Haiyan Li, Chaopeng Hong, Yu Lei, Yawen Kong, Qiang Zhang, Kebin He

**Affiliations:** ^1^ Shenzhen Key Laboratory of Ecological Remediation and Carbon Sequestration, Institute of Environment and Ecology, Tsinghua Shenzhen International Graduate School, Tsinghua University, Shenzhen 518055, China.; ^2^ State Environmental Protection Key Laboratory of Sources and Control of Air Pollution Complex, Beijing 100084, China.; ^3^ Laboratoire des Sciences du Climat et de l’Environnement, LSCE/IPSL, CEA-CNRS-UVSQ, Université Paris-Saclay, Gif-sur-Yvette, France.; ^4^ Department of Meteorology and Air Quality, Wageningen University, Wageningen, Netherlands.; ^5^ Climate Observations Department, Royal Netherlands Meteorological Institute, De Bilt, Netherlands.; ^6^ Department of Energy, Environmental and Chemical Engineering, Washington University, St. Louis, MO, USA.; ^7^ Department of Physics and Atmospheric Science, Dalhousie University, Halifax, NS, Canada.; ^8^ R&D Satellite Observations, Royal Netherlands Meteorological Institute (KNMI), De Bilt, Netherlands.; ^9^ School of Civil and Environmental Engineering, Harbin Institute of Technology, Shenzhen 518055, China.; ^10^ Chinese Academy of Environmental Planning, Beijing 100012, China.; ^11^ Ministry of Education Key Laboratory for Earth System Modeling, Department of Earth System Science, Tsinghua University, Beijing 100084, China.; ^12^ State Key Joint Laboratory of Environment Simulation and Pollution Control, School of Environment, Tsinghua University, Beijing 100084, China.

## Abstract

Response actions to the coronavirus disease 2019 perturbed economies and carbon dioxide (CO_2_) emissions. The Omicron variant that emerged in 2022 caused more substantial infections than in 2020 and 2021 but it has not yet been ascertained whether Omicron interrupted the temporary post-2021 rebound of CO_2_ emissions. Here, using satellite nitrogen dioxide observations combined with atmospheric inversion, we show a larger decline in China’s CO_2_ emissions between January and April 2022 than in those months during the first wave of 2020. China’s CO_2_ emissions are estimated to have decreased by 15% (equivalent to −244.3 million metric tons of CO_2_) during the 2022 lockdown, greater than the 9% reduction during the 2020 lockdown. Omicron affected most of the populated and industrial provinces in 2022, hindering China’s CO_2_ emissions rebound starting from 2021. China’s emission variations agreed with downstream CO_2_ concentration changes, indicating a potential to monitor CO_2_ emissions by integrating satellite and ground measurements.

## Teaser

Omicron affected most of populated and industrial provinces in China in 2022, which cut more CO_2_ emissions than the 2020 lockdown.

## INTRODUCTION

In response to the public health concerns about the coronavirus disease 2019 (COVID-19), stringent confinement measures were implemented in many countries, which temporally cut emissions of carbon dioxide (CO_2_) and air pollutants due to restricted mobility and economic activities (*1*, *2*). For example, the reduction in CO_2_ emissions from China, where the virus outbreak and city lockdown were first reported in Wuhan (*3*), was estimated to have reached 2.6 to 11.5% (*4*–*6*) between January and April 2020 compared to the same months in 2019. Global daily fossil CO_2_ emissions were estimated to have decreased by 17% by April 2020 (*4*). After the first COVID wave in 2020, a rebound of CO_2_ emissions emerged with the lift of lockdown measures and the recovery in energy demand which was spurred by economic stimulus packages in China and other countries (*7*–*9*). These dynamics of CO_2_ emissions reflect the immediate response of our society to global pandemic-induced economic disruptions, illustrating the nexus among energy, society, and emissions and their interactive impacts on climate (*10*, *11*).

Two years after the COVID-19 outbreak, the Omicron variant appeared in late 2021, and the fast spreading of this variant outstripped common virus control measures (*12*). In China, Omicron affected the most populated and economically developed cities (e.g., Beijing, Shanghai, Guangzhou, Shenzhen, and Xi’an) from the east coast to the western provinces, which was distinct from the first wave in early 2020 when the provinces surrounding Wuhan were most affected by the virus. The outbreak of Omicron in the economically most developed cities led to stringent lockdowns in China in early 2022, and the policy responses kept evolving to minimize the lockdown areas and periods (*13*). Since Omicron affected the economic heart of eastern China in 2022, China’s gross domestic product (GDP) increased by only 0.4% in the second quarter of 2022, while Shanghai’s GDP experienced an unprecedented decline of 13.7% (www.stats.gov.cn/). Thermal power generation and cement production decreased by 1.8 and 14.8%, respectively, between January and April 2022 compared to the same months in 2021 (www.stats.gov.cn/). Yet, the changes in China’s CO_2_ emissions in early 2022 compared with the first COVID wave in early 2020, with the sectoral, regional, and economic drivers, have not been reported quantitatively.

The capacity to track emissions in near real time has developed quickly during the COVID-19 pandemic. The bottom-up approach, which relies on high-resolution, timely, and sectoral activity data and emission factors, has been developed to construct multi-resolution emission inventories with low time latency (*14*–*16*). The availability of reliable, transparent, and timely activity data is essential to developing such near–real-time emission inventories. In China, because of a lack of sufficient data, temporal profiles derived from limited sectors and regions, e.g., electricity generation, natural gas consumption, and traffic volume in certain areas, had to be used to represent emission sources throughout the country, which misrepresented emission variations of specific sectors for which detailed and timely updated information was not accessible (*16*). Supplementary to the bottom-up method, the top-down approach infers emissions from atmospheric observations of species abundance (e.g., satellite- and ground-based measurements) (*17*–*20*). The reductions of CO_2_ concentrations in the troposphere due to lockdowns can be detected from observations to a certain extent (*21*–*23*) while could be quite challenging from space (*24*). The sparse sampling of CO_2_ by current satellites and in situ networks, as well as the high background concentrations of CO_2_ associated with the large spatial-temporal variations due to natural fluxes, hinders an accurate quantification of regional CO_2_ anthropogenic emissions in China (*25*).

In our previous study (*6*), we inferred the decline and rebound of China’s CO_2_ emissions in early 2020 from nitrogen dioxide (NO_2_) column observations which were retrieved from the TROPOspheric Monitoring Instrument (TROPOMI) satellite, with an inversion system that integrated both bottom-up and top-down information. The bottom-up data provided prior emission maps by source sector, and the top-down model optimized the changes in nitrogen oxide (NO_x_) emissions from the TROPOMI NO_2_ column and further constrained the dynamics of sectoral NO_x_ and CO_2_ emissions. Here, we update and improve this method to develop prior emission maps with more sector-specific, accurate activity data and use a new version of the TROPOMI NO_2_ that reduces the underestimation bias in NO_2_ retrievals and provides consistent NO_2_ column series since 2018 (see Materials and Methods). With the updated inversion system and improved input datasets, we estimate the 10-day moving average, sector-specific, provincial NO_x_, and CO_2_ emissions in China between January and April from 2020 to 2022. The dynamics in NO_x_ and CO_2_ emissions in early 2022 are analyzed by sector and by the province to resolve the drivers behind the changes in emissions compared to the first wave of COVID in early 2020.

## RESULTS

### Satellite reveals a larger drop in China’s emissions in 2022 than in 2020

**Fig. 1.**
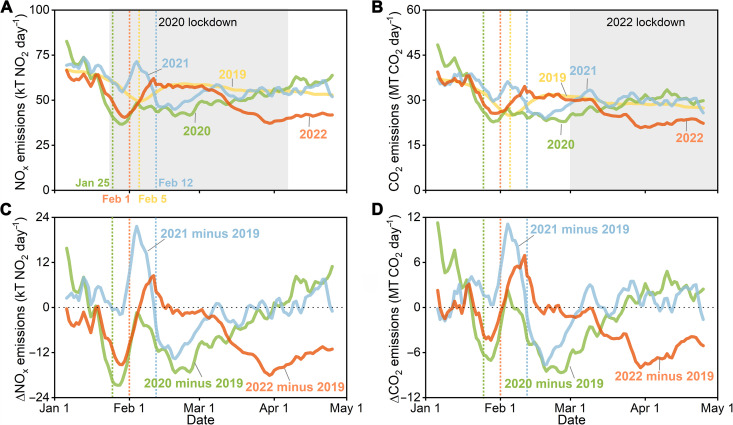
Ten-day moving average NO_x_ and CO_2_ emissions of China from January to April in 2019, 2020, 2021, and 2022. The 2019 emissions are derived from the MEIC (Multi-resolution Emission Inventory for China) emission inventory model, and the emission results from 2020 to 2022 are derived from the TROPOMI-constrained inversion estimates. (**A**) presents the 10-day moving average NO_x_ emissions and (**C**) presents the changes in NO_x_ emissions from 2019 to 2020, 2021, and 2022, respectively. (**B** and **D**) are plotted for CO_2_ as (A) and (C). The vertical dashed lines refer to the Chinese Spring Festival Day in different years. The gray shadings in (A) and (B) correspond to the 2020 lockdown and the 2022 lockdown periods, respectively.

The 2019 emissions represent the pre-COVID levels, which serve as the baseline to understand the response of China’s emissions to the COVID-induced restrictions in 2020 and 2022. The total NO_x_ emissions between January and April in China dropped by 7.2 and 12.5% in 2020 (green curves in Fig. 1, A and C) and 2022 (red curves in Fig. 1, A and C), respectively, compared to those in 2019 (yellow curve in Fig. 1A). The inferred CO_2_ emissions declined by 2.4 and 6.5% in 2020 (green curves in Fig. 1, B and D) and 2022 (red curves in Fig. 1, B and D), respectively, compared to the 2019 emissions (yellow curve in Fig. 1B). The 2021 emissions of NO_x_ and CO_2_ (blue curves in Fig. 1) were slightly higher than the 2019 emissions by less than 3%. The consistent interannual variation between CO_2_ emissions and industrial production records on a monthly scale qualitatively demonstrates the robustness of our estimation (fig. S1). The decline in CO_2_ emissions by 2.4% between January and April from 2019 to 2020 is close to the bottom-up estimate by a global emission model (2.6%) (*4*), but lower than our previous estimate of 11.5% (*6*), mainly due to the use in this study of the latest, low-bias corrected TROPOMI NO_2_ column retrievals (processor version 2.3.1). With our current inversion system, the TROPOMI NO_2_ columns of the previous version (processor version 1.3), which was used in our previous study (*6*), can reproduce the temporary decline in CO_2_ emissions but give a larger reduction of 9.7% between January and April 2020 (table S1). The slightly smaller estimate than our previous results (*6*) is due to the different prior emissions developed for the update of the inversion system.

A drop in national NO_x_ and CO_2_ emissions of comparable magnitude was recurrent during the Chinese Spring Festival holidays from 2019 to 2022 (Fig. 1 and fig. S2), while large discrepancies in emission decline were observed during the COVID lockdown periods between 2020 and 2022. In 2020, the COVID-19 case surge was concentrated in Wuhan and the nearby regions, with the maximum amount of national daily new cases approaching 16,000 (fig. S3), which triggered a stringent lockdown from 23 January 2020 that lasted for more than 2 months in China (gray shading in Fig. 1A, denoted as the 2020 lockdown). During this period, the cumulative emissions of China’s NO_x_ and CO_2_ declined by 14.4 and 9.0%, respectively, compared to the corresponding period in 2019. After April 2020, China’s emissions rebounded and surpassed the pre-lockdown levels with the lockdown easing. In 2021, much fewer COVID-19 new cases (less than 200 per day) were detected in China; thus, there was no large-scale lockdown, and the NO_x_ and CO_2_ emissions were not disturbed compared to 2019 (fig. S3).

In 2022, the daily confirmed COVID-19 cases, mainly the Omicron variant, emerged in March and approached a maximum of approximately 6000 new cases per day (fig. S3), which led to widespread restrictions and lockdowns in the economically developed and populated cities (e.g., Beijing, Shanghai, Guangzhou, and Shenzhen). Since there was no coordinated lockdown across different cities, our study regards March and April as the lockdown period of 2022 (gray shading in Fig. 1B), when the COVID-19 cases rose sharply and the emissions of CO_2_ and NO_x_ became substantially lower than those in 2019 (fig. S3) as diagnosed by atmospheric observations. During this lockdown period in 2022, China’s NO_x_ and CO_2_ emissions declined by 19.9 and 15.0%, respectively, compared to the 2019 emission levels. After April 2022, the lockdown continued in several of China’s cities (e.g., Shanghai reopened on 1 June), with emissions remaining at a low level and not rebounding as after April 2020. The greater emission reductions during the 2022 lockdown than the 2020 lockdown, which is shown by our inversions, are corroborated by the substantial decline in the satellite-observed NO_2_ column densities (fig. S4). The surface stations downwind of China (fig. S5) also observed substantial decreases in CO_2_ concentration enhancement above the background levels within air parcels from China in 2022 (fig. S6), which reflects a steep and lasting CO_2_ emission decline (see Materials and Methods). Since different ground stations captured air parcels passing over different provinces in China, we also observe broadly consistent variations in CO_2_ concentration enhancement and the corresponding regional CO_2_ emissions despite uncertainties still existing (see Materials and Methods).

### Sectoral emission reductions during the lockdown in 2020 and 2022

**Fig. 2.**
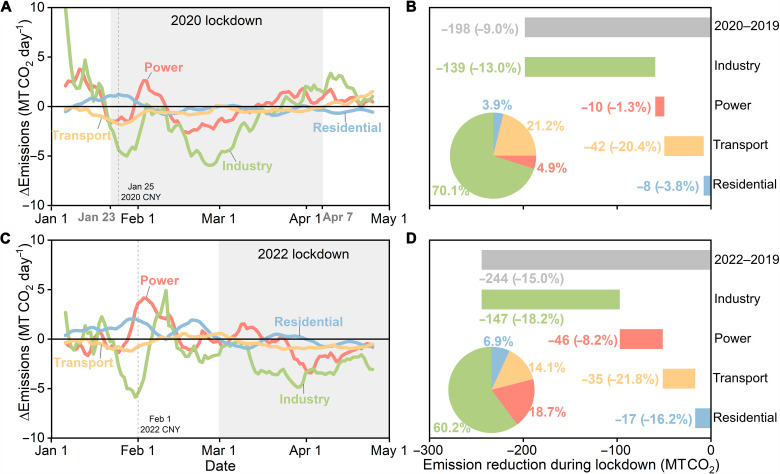
Changes in the sectoral CO_2_ emissions of China in 2020 and 2022 compared to those in 2019. The 10-day moving average emission changes are shown for the power, industrial, transport, and residential sectors from 2019 to 2020 (**A**) and from 2019 to 2022 (**C**). The sector-specific CO_2_ emission reductions during the 2020 lockdown [gray shading in (A)] and the 2022 lockdown periods [gray shading in (C)] are presented in (**B** and **D**), respectively. The numbers in (B) and (D) show the absolute emission reductions and relative changes in emissions (in brackets) for each sector. The pie charts show the contribution of each sector to the total emission reductions during the lockdown periods.

The larger emission reductions during the lockdown in 2022 compared with 2020 are mainly attributed to the power and industry sectors (CO_2_ shown in Fig. 2 and fig. S7 and NO_x_ shown in figs. S8 and S9), while the transport sector exhibited comparable emission reductions between 2020 and 2022. The reductions in the industry and power CO_2_ emissions were estimated to have reached 18.2% [equivalent to 147 million metric tons (MT) CO_2_; green bar in Fig. 2D] and 8.2% (46 MT CO_2_; red bar in Fig. 2D), respectively, during the 2022 lockdown, compared to the corresponding periods in 2019. These emission reductions are larger than the emissions drop during the 2020 lockdown when the industry and power sectors reduced their CO_2_ emissions by 13.0% (139 MT CO_2_; green bar in Fig. 2B) and 1.3% (10 MT CO_2_; red bar in Fig. 2B), respectively. Since industry uses more than two-thirds of the electricity in China, the larger drop in industrial production and emissions in 2022 than in 2020 led to a more substantial decline in the electricity generation and CO_2_ emissions from the power sector, which occurred from late March to April in 2022 based on our 10-day moving average inversion emission results (red curve in Fig. 2C).

The inversion-estimated larger decline in industrial and power emissions in 2022 than in 2020 is consistent with the monthly statistics of industrial production which can reflect the variation of energy consumption and emissions with time. The productions of steel and cement in China in April 2022 (the second month of the 2022 lockdown) were 5.2 and 18.9%, respectively, lower than those in April 2021, while the productions in March 2020 (the second month of the 2020 lockdown) were 1.7 and 18.3% lower than those in March 2019. The thermal power generation in China in April 2022 was 11.8% lower than that in April 2021, while the decrease in March 2020 was only 7.5% compared to that in March 2019. Since the year-on-year monthly growth rates were based on the adjusted statistical caliber of each year, it could be difficult to directly compare the monthly industrial production in 2022 with that in 2019 due to a lack of consistent monthly statistics data. The annual production in 2021 and 2019 was close to each other, which can provide us with an approximately equivalent comparison basis.

The industry and power sectors accounted for more than three-quarters of the reductions in China’s CO_2_ emissions during the lockdown in both 2020 and 2022 (Fig. 2), which reflects the fact that these two sectors dominated China’s CO_2_ emissions and were largely affected by the COVID-19 lockdown measures. The 2022 lockdown was mainly enforced after the Chinese Spring Festival holiday when people returned to work and industrial production tended to rebound to normal levels. However, the 2020 lockdown overlapped with the Spring Festival holiday in 2020 when industrial production already stayed at a low level due to the low market demand during the holiday. This could partly explain why the 2022 lockdown due to the Omicron variant cut down China’s industrial production and the related CO_2_ emissions more substantially than the 2020 lockdown during the first COVID wave.

For the residential sector, the CO_2_ emissions during the 2022 lockdown declined by 16.2% (17 MT CO_2_; blue bar in Fig. 2D), which is larger than the emission reduction of 3.8% (8 MT CO_2_; blue bar in Fig. 2B) during the 2020 lockdown. This is probably due to lower demand for residential heating in the winter and spring of 2022, which is reflected by 6.2% lower heating degree days during the 2022 lockdown period than the corresponding time in 2019. Overall, the residential sector only accounted for nearly 6% of China’s CO_2_ emissions; therefore, it had little impact on the inter-annual variation of total emissions.

### Provinces most affected by CO_2_ emission reductions during lockdown

**Fig. 3.**
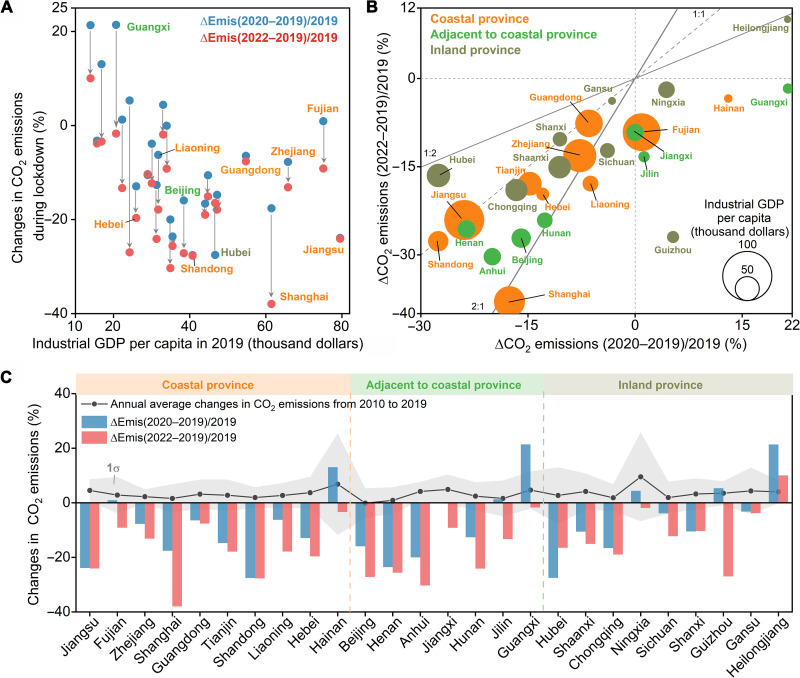
Comparison of provincial CO_2_ emission changes during the lockdown periods in 2020 and 2022. (**A**) Analysis of provincial CO_2_ emission changes during the 2020 lockdown (blue dots) and the 2022 lockdown (red dots), and the industrial GDP per capita of each province. (**B**) Comparison of provincial CO_2_ emission changes during the lockdowns in 2020 and 2022. The color of each dot represents the type of province and the size of each dot represents the industrial GDP per capita of each province. (**C**) Comparison of provincial CO_2_ emissions changes during the lockdown periods in 2020 and 2022 from inversion estimates and the annual average changes in CO_2_ emissions from 2010 to 2019 (black curve) from the MEIC model. We only plot the provinces where TROPOMI NO_2_ tropospheric vertical column densities larger than 1 × 10^15^ molecules/cm^2^ can cover more than 80% of their anthropogenic NO_x_ emissions.

Provinces with larger industrial GDP tended to reduce more CO_2_ emissions during the lockdown in both 2020 and 2022 (Fig. 3, A and B), which reflects the dominant role of industry in CO_2_ emission reduction. The inversion estimated reductions in provincial emissions are broadly consistent with the decline in TROPOMI NO_2_ columns across provinces (fig. S10). The reductions in CO_2_ emissions during the lockdowns in 2020 and 2022 largely exceeded the interannual variation of emissions between 2010 and 2019 for most of the provinces (Fig. 3C), indicating a substantial short-term effect of the COVID-19 lockdown on provincial CO_2_ emissions. Comparing the lockdown periods in 2022 with 2020, the provinces with larger industrial GDP showed a sharper drop in CO_2_ emissions during the 2022 lockdown than the 2020 lockdown, especially the provinces on the east coast of China (e.g., Shanghai, Guangdong, Zhejiang, Fujian, Hebei, and Liaoning; the orange ones in Fig. 3, A and B) and the adjacent provinces (e.g., Hunan, Anhui, and Jiangxi; the green ones in Fig. 3, A and B). However, the inland provinces in China (the gray dots in Fig. 3B) tended to present broadly comparable reductions in CO_2_ emissions between the 2022 lockdown and the 2020 lockdown, such as in Shanxi, Shaanxi, Gansu, and Chongqing.

For the provinces located far away from Wuhan (at the first and fourth quadrants of Fig. 3B), cumulative CO_2_ emissions slightly grew during the Wuhan lockdown in 2020, such as in Heilongjiang, Guangxi, Hainan, Ningxia, Guizhou, and Jilin. These provinces had shorter lockdown periods than Wuhan in 2020; thus, their economy and CO_2_ emissions were not disturbed substantially. However, during the 2022 lockdown, all of these provinces except Heilongjiang reduced their CO_2_ emissions due to the impacts of stringent lockdowns induced by Omicron on the economy. For example, the Jilin province reported nearly 40 thousand cases during January and April 2022, 400 times larger than the total number of cases (less than 100) between January and April 2020, which triggered a stringent lockdown of about 2 months. For Heilongjiang, the inversion estimated increase in CO_2_ emissions by 10.0% in 2022 corresponds to the growing Manufacturing Value Added (MVA) reported by this province, which increased by 10.3% in March 2022 due to fewer Omicron cases and the less lockdown influence.

The reductions in provincial CO_2_ emissions reveal the spatial disparity of lockdown impacts between 2020 and 2022, corresponding to the different transmission features of the Omicron variant. Our inversion results suggest that the CO_2_ emissions from China’s coastal provinces (orange dots in Fig. 3B) where industry contributes a larger share of GDP, were more affected by the restrictions and lockdowns in 2022 than those in 2020 (fig. S11). In 2020, the leading region for COVID lockdown and emission reductions mainly concentrated on the Hubei province, where Wuhan is located, and the surrounding industrialized provinces such as Anhui, Shandong, and Jiangsu (fig. S11, A and B). The impacts on economic activities and CO_2_ emissions from the 2020 lockdown were thus limited in space and time. However, the Omicron variant in early 2022 severely affected the provinces on the east coast of China, which accounted for more than half of China’s industrial production. The intense virus transmission over these populated and economically developed provinces led to widespread lockdowns and subsequent emission reductions in 2022. Since the coastal provinces play a key role in the import, manufacturing, and export of China’s economies, the impacts of the COVID lockdown spilled over into the other provinces and made a widespread impact on CO_2_ emission reduction. This could explain the larger percentage decrease in CO_2_ emissions over most of China’s provinces during the lockdown in 2022 (fig. S11C), although the COVID-19 cases increased sharply mainly over the coastal provinces (fig. S11D).

## DISCUSSION

Our inversion estimates of NO_x_ and CO_2_ emissions are subject to systematic and random errors underlying the system configurations and data input, which are inevitable but have been reduced in this study as much as possible to lessen the likelihood of error influence on our main conclusions. We mainly focused on the relative changes in TROPOMI NO_2_ columns and the inversion estimates of NO_x_ and CO_2_ emissions across different years, which can reduce a major part of the systematic errors in the analysis. The GEOS-Chem model was used to build the relation between relative changes in NO_x_ emissions and NO_2_ columns and to remove the meteorological variation-induced relative changes of NO_2_ columns. The relative changes used here are expected to cancel out a large part of the systematic bias of GEOS-Chem. Besides, we analyzed the temporal variation of emissions based on spatial and sectoral aggregates, which are expected to minimize random errors. Our previous study addressed most of the systematic and random errors in the inversion system (*6*). In this study, we further evaluated our results through sensitivity tests (see Materials and Methods) based on different assumptions of emission factor trends (fig. S12), different versions of TROPOMI NO_2_ retrievals (figs. S13 and S14), and different scale factors linking changes in NO_x_ emissions with NO_2_ columns (see Materials and Methods). The sensitivity inversions show that the results of this study are robust, which indicates a substantial decline in CO_2_ emissions in early 2022 due to the COVID lockdown, larger than the emission drop during the lockdown of the first wave in 2020.

Even after 2 years of the COVID outbreak, the transmission of the Omicron variant still had a large impact on China’s anthropogenic emissions because the populated and economically developed regions were more affected by the fast-spreading Omicron variant. The leading provinces that reduced CO_2_ emissions were mainly located on the east coast of China in 2022. Since these provinces accounted for more than half of China’s industrial production and CO_2_ emissions, the response of CO_2_ emissions to the COVID restrictions and socio-economic stimulus packages thus had broad implications for our climate. When and how fast the CO_2_ emissions could rebound in 2022 and afterward need to be monitored continuously and accurately with low latency based on scientific tools. Compared to our previous study which estimated the emissions only in early 2020, this study demonstrates that our inversion system can track the emission dynamics across different years. Satellite observations of reactive trace gasses in the troposphere, combined with an inversion system with a translation algorithm to CO_2_ emissions by sector, could contribute to the global carbon monitoring framework from the top-down perspective in the future.

## MATERIALS AND METHODS

Our method is mainly based on the atmospheric inversion framework developed by our previous work (*6*). We first update China’s NO_x_ and CO_2_ emissions to the period 2020–2022 based on the MEIC (Multi-resolution Emission Inventory for China) inventory in 2019 using the bottom-up approach (*16*). Second, we infer the 10-day moving average of NO_x_ total emissions from TROPOMI NO_2_ column retrievals using the mass balance method (*26*, *27*). Last, we constrain the sectoral (i.e., power, industry, residential, and transportation) NO_x_ emissions by correcting the bottom-up prior emission maps based on the inversion-based reconstruction of NO_x_ emission distributions. The sectoral CO_2_ emissions are further estimated on the basis of the inversion-constrained sectoral NO_x_ emissions and sector-specific CO_2_-to-NO_x_ emission ratio maps. Please refer to our previous study for more details. The improvements compared to our previous approach include the refinement of bottom-up emission updates with more detailed activity data and the adoption of the latest, low-bias corrected TROPOMI NO_2_ column retrievals (processor version 2.3.1). Here, we briefly introduce the methods and mainly focus on the improvement of our updated system.

### Bottom-up estimation of prior NO_x_ and CO_2_ emissions

We estimate China’s monthly, gridded emissions of NO_x_ and CO_2_ between 2020 and 2022 using the bottom-up approach (*16*) based on the 2019 emissions from the MEIC model and the year-over-year monthly changes in activity data and emission factors.

More sectoral activity data proxies are used in this study than in our previous study to better represent the monthly variations of emissions by sector (i.e., power, industry, residential, and transport). The monthly growth rates of power, cement, and iron production, as well as the manufacturing value-added, are used to represent the monthly variation of activity data of the power and industry sectors. Since the statistics data for January and February are reported together, we divide them into each month based on the monthly production index, which is more reasonable than our previous study assuming that the activities of January and February follow the same growth rate. The monthly activity data are further split into daily data on the basis of the daily profiles derived from continuous emission monitoring systems (*28*), which is more accurate than the national plant operating rates used before. For the residential sector, we use population-weighted heating degree days to represent the monthly and daily changes in provincial residential energy use, except in Guangdong, Guangxi, and Hainan where domestic heating is not necessary during the winter season. The residential activity data from these three provinces are not changed. For the transport sector, the monthly changes in on-road cargo turnover and construction area are used to represent the variations in on-road and off-road activity data, respectively, while our previous study did not differentiate between on-road and off-road activities. The daily profiles of the transport sector are derived from the Baidu migration data (https://qianxi.baidu.com) for each city in China.

The emission factors of CO_2_ are dependent on fuel carbon content, heating value, and oxidation rate, each of which is assumed unchanged for each fuel type; therefore, CO_2_ emission factors are not changed since 2019. The NO_x_ emission factors have been declining in China due to air pollution control actions since 2013 (*29*, *30*). Simplified assumptions are made to extrapolate the decreasing trends of NO_x_ emission factors from 2019 to 2022 based on the progress of air pollution control in China. For the power sector, approximately 90% of the coal-fired units in China met the ultralow emission standard by 2020, and the coal-fired units with a capacity lower than 20 GW have been phased out (*31*). The emission reduction potential has already been realized almost completely; therefore, the NO_x_ emission factors of the power sector are assumed unchanged from 2020. Besides, China’s clean air actions phased out 0.44 billion tons of polluted cement manufacturing capacities, 0.42 billion tons of iron manufacturing capacities, and 25 million dirty old vehicles by 2020 (*30*, *32*). Since pollution control is still in progress for the industry and transport sectors, we assume that the percentage decrease in NO_x_ emission factor is half of that during the last year, which is based on the MEIC emission model estimates since 2017 (*16*, *29*). The slowing downward trend of NO_x_ emission factors approximates a gradual realization of emission mitigation potential. We perform two sensitivity tests to evaluate the influence of the assumed different trends of NO_x_ emission factors on the inversion estimates of NO_x_ and CO_2_ emissions (table S2).

### Top-down estimation of NO_x_ emissions based on TROPOMI NO_2_

We infer anthropogenic NO_x_ emissions from the TROPOMI NO_2_ columns on a 10-day moving average scale using the mass balance method (*26*, *27*), which assumes a localized relation between the changes in NO_2_ tropospheric vertical column densities (TVCDs) and anthropogenic NO_x_ emissions.

$$E_{t,i,\mathrm{TROPOMI},y}=\left[1+\beta_{t,i}{\left(\frac{\text{Δ}\text{Ω}}{\text{Ω}}\right)}_{t,i,\mathrm{anth},y}\right]\times E_{t,i,\mathrm{bottom}\text{-}\mathrm{up},2019}$$ (1)

$${\left(\frac{\text{Δ}\text{Ω}}{\text{Ω}}\right)}_{t,i,\mathrm{anth},y}=\frac{{\text{Ω}}_{t,i,\mathrm{sate},y}}{{\text{Ω}}_{t,i,\mathrm{sate},2019}}-\frac{{\text{Ω}}_{t,i,\mathrm{simu}\_\mathrm{fixemis},y}}{{\text{Ω}}_{t,i,\mathrm{simu},2019}}$$ (2)

where *t*, *i*, and *y* represent the 10-day window, the model grid cell (i.e., 0.5° × 0.625°), and the year (i.e., 2020, 2021, and 2022), respectively. *E*_*t*,*i*,TROPOMI,y_ is the anthropogenic NO_x_ emissions constrained by TROPOMI NO_2_ TVCDs. *E*_*t*,*i*,bottom-up,2019_ is the anthropogenic NO_x_ emissions in 2019 derived from the MEIC model. β_*t*,*i*_ is a unitless factor that relates the changes in NO_2_ TVCDs to changes in anthropogenic NO_x_ emissions alone (*26*). (∆Ω/Ω)_*t*,*i*,anth,*y*_ estimates the relative changes in NO_2_ TVCDs due to anthropogenic NO_x_ emissions from 2019 to 2020, 2021, and 2022, respectively. (∆Ω/Ω)_*t*,*i*,anth,*y*_ is calculated on the basis of the difference between Ω_*t*,*i*,sate,*y*_/Ω_*t*,*i*,sate,2019_ and Ω_*t*,*i*,simu_fixemis,*y*_/ Ω_*t*,*i*,simu,2019_. Ω_*t*,*i*,sate,*y*_/Ω_*t*,*i*,sate,2019_ represents the relative differences in TROPOMI NO_2_ TVCDs, and Ω_*t*,*i*,simu_fixemis,*y*_/Ω_*t*,*i*,simu,2019_ represents the relative differences in NO_2_ TVCDs due to meteorological factors, which are derived from chemical transport model simulations with the fixed 2019 emissions.

The TROPOMI level-2 NO_2_ columns between January and April from 2019 to 2021 are derived from the Sentinel-5P Product Algorithm Laboratory (S5P-PAL) (https://data-portal.s5p-pal.com/products/no2.html), which processes the NO_2_ retrievals using the latest operational processor (version 2.3.1). The TROPOMI NO_2_ columns in 2022 are derived from the official offline processing retrievals of version 2.3.1 (www.temis.nl/airpollution/no2col/no2regio_tropomi.php). The processor version 2.3.1 adopts a wider range of wavelengths in the O_2_-A band compared to previous retrieval data versions, leading to a decrease in cloud pressure and an associated increase in tropospheric NO_2_ columns (*33*, *34*). This new processor reduces the underestimation bias of TROPOMI NO_2_ columns, especially over polluted regions. We observe 10 to 40% larger NO_2_ columns than those of version 1.3 over China between January and April in 2019 and 2020 (fig. S13), in line with the previous study (*34*).

β is estimated for each grid cell on the basis of the GEOS-Chem model simulation by perturbation of anthropogenic NO_x_ emissions. We use GEOS-Chem 12.3.0 (https://geoschem.github.io/) driven by the meteorological fields from the MERRA-2 Reanalysis of the NASA Global Modeling and Assimilation Office (*35*). Detailed settings of the GEOS-Chem model were described in our previous work (*6*). β is calculated on the basis of the perturbation factor (i.e., −40%) of China’s NO_x_ emissions divided by the corresponding relative changes in simulated NO_2_ columns, which are based on the simulations for 2019. Ω_*t*,*i*,simu_fixemis,*y*_ refers to the NO_2_ columns simulated with the fixed anthropogenic emission in 2019 driven by MERRA-2 meteorological fields in year *y* using GEOS-Chem. Therefore, Ω_*t*,*i*,simu_fixemis,*y*_/Ω_*t*,*i*,simu,2019_ reflects the changes in NO_2_ TVCDs resulting only from the meteorological fields in different years. We further perform sensitivity tests that vary β by ±20% to evaluate the impact of the uncertainties of β on emission estimation. The results suggest that the main conclusion of this study—emission reductions were larger in early 2022 than those in 2020—is not changed. For example, the NO_x_ emissions from Shanghai were estimated to have declined by 32.7 to 49.1% during the 2022 lockdown compared to the same period in 2019 corresponding to the ±20% perturbation of β, which is larger than the emission reductions of 20.1 to 30.9% during the 2020 lockdown.

### Estimation of NO_x_ and CO_2_ emissions by source sector

The TROPOMI-constrained NO_x_ emissions are used to correct bottom-up NO_x_ emissions by source sector on the basis of the differences in emissions over the grid cells dominated by different source sectors, the method of which was described in our previous study (*6*). The sectoral NO_x_ emissions are further transformed to estimate CO_2_ emissions on the basis of the sector-specific CO_2_-to-NO_x_ emission ratios.

$$C_{s,t,i,\mathrm{TROPOMI},y}=E_{s,t,i,\mathrm{TROPOMI},y}\times \frac{{{EF_{\mathrm{CO}}}_{2}}_{s,i,\mathrm{bottom}\text{-}\mathrm{up},2019}}{{{EF_{\mathrm{NO}}}_{x}}_{s,i,\mathrm{bottom}\text{-}\mathrm{up},2019}\times (1-r{\mathrm{NO}}_{xs,i,y})}$$ (3)

where *s* represents the sector. *C*_*s*,*t*,*i*,TROPOMI,*y*_ and *E*_*s*,*t*,*i*,TROPOMI,*y*_ are CO_2_ and NO_x_ emissions from sector *s*, respectively. *EF*_CO2*s*,*i*,bottom-up,2019_ and *EF*_NOx*s*,*i*,bottom-up,2019_ are the average emission factors of CO_2_ and NO_x_ in 2019, respectively, both of which are derived from the MEIC emission model. *r*NO_x*s*,*i*,*y*_ is the percentage decrease of NO_x_ emission factor from 2019 to the year *y*, which is estimated using the bottom-up approach described in the “Bottom-up estimation of prior NO_x_ and CO_2_ emissions” section.

On the basis of the MEIC model, the CO_2_-to-NO_x_ emission ratios in 2019 vary by source sector due to different combustion and pollution control techniques. The power and transport sectors have the largest and smallest CO_2_-to-NO_x_ emission ratios, respectively, because NO_x_ pollution control measures are substantially deployed by the power sector, while oil used in the transport sector has low carbon content but large emission factors of NO_x_. The CO_2_-to-NO_x_ emission ratios are estimated to have slightly increased since 2019 because of the decreasing NO_x_ emission factors due to pollution control in the industrial and transport sectors (fig. S15). The drivers of daily variations in CO_2_-to-NO_x_ emission ratios are partly attributed to the impacts of the COVID-19 lockdown. For example, the activities and emissions of the transport sector are most influenced by the lockdown restrictions, we thus observe a large increase in CO_2_-to-NO_x_ emission ratios during the lockdown periods in 2020 and 2022 (fig. S15).

### Evaluation of the impacts of TROPOMI NO_2_ retrievals and NO_x_ emission factors

To investigate the influences of TROPOMI NO_2_ retrievals on emission estimation, we assimilate the NO_2_ columns of processor version 1.3 into our updated inversion system and compare the emission results with those constrained by the NO_2_ columns of processor version 2.3.1 (table S1). The TROPOMI version 1.3 was used in our previous inversion system, and the associated estimates of CO_2_ emission reductions (11.5%) in the first 4 months of 2020 are much larger than the bottom-up estimate by a global emission model (2.6%) (*4*). On the basis of the TROPOMI version 1.3, our updated inversion system in this study also suggests that China’s CO_2_ emissions between January and April declined largely by 9.7% from 2019 to 2020, which is larger than the TROPOMI version 2.3.1–based inversion estimates (2.4%). The TROPOMI version 2.3.1 has reduced the bias of NO_2_ retrievals over polluted regions, and the updated estimate of CO_2_ emission reductions in early 2020 is close to the estimate by a previous global emission inventory study (*4*). The discrepancy in emission estimates based on different TROPOMI NO_2_ versions suggests that our inversion system benefits from the improvement of satellite observations.

The CO_2_-to-NO_x_ emission ratios play an important role in the estimate of CO_2_ emissions based on NO_x_ inversions. We perform two sensitivity inversion experiments that vary the downward trends of NO_x_ emission factors of the industrial and transport sectors in the bottom-up inventories. One experiment assumes that NO_x_ emission factors unchanged since 2020, while the other one depicts an exponential decrease in NO_x_ emission factors in the years since 2020. These two experiments represent two extreme scenarios for NO_x_ pollution control in China (table S2). The inversion results suggest that the reduction in CO_2_ emissions during the lockdown in 2022 spans from −15.9 to −13.5% (equivalent to −259.0 to 219.0 MT CO_2_) compared to the corresponding time in 2019, which is larger than the CO_2_ emission reductions during the lockdown period in 2020 (−198.4 MT CO_2_) (fig. S12). Therefore, the potential variations in NO_x_ emission factor trends, as well as the associated CO_2_-to-NO_x_ emission ratios, do not largely influence the main conclusion of this study, which suggests a more substantial reduction in CO_2_ emissions during the lockdown period in 2022 than that during the 2020 lockdown period.

### Evaluation of CO_2_ emission variations based on surface CO_2_ observations

The long lifetime of CO_2_ makes it hard to directly investigate the effect of its emission variation on CO_2_ concentration (*36*). On the basis of a well-established method (*37*), we calculate CO_2_ concentrations above the background levels (ΔCO_2_) using the CO_2_ concentrations from four surface stations (fig. S5), including Anmyeondo (AMY), Jeju Gosan (JGS), Ryori (RYO), and Yonagunijima (YON), all of which are located downwind of China (World Data Centre for Greenhouse Gases, https://gaw.kishou.go.jp/). The Minamitorishima (MNM) at the remote ocean of a similar latitude band is used as the background station, whose observed concentrations are subtracted from the observations of AMY, JGS, RYO, and YON to calculate ΔCO_2_. Only the hourly observations between 12:00 and 17:00 of local time (when the transport is dynamic) are used in the ΔCO_2_ calculation to minimize the influence of local emission sources. We further select the observations whose backward trajectories mainly pass over China based on 7-day backward trajectory simulations starting at 15:00 using Meteoinfo (www.meteothink.org/). Our analysis was performed between January and May from 2015 to 2022, with the 2015–2019 average representing the pre-COVID levels.

The sign and magnitude of ΔCO_2_ changes observed by each surface station are well explained by the inversion-estimated CO_2_ emissions from the regions whose CO_2_ plumes are measured (fig. S6). The AMY and JGS stations mainly observed air parcels from North China, where the reduction of ΔCO_2_ was larger in 2022 than in 2020, and ΔCO_2_ in 2021 was higher than that in the 2015–2019 mean. These are consistent with the inter-annual changes in CO_2_ emissions from North China derived from our inversions (fig. S11). The RYO station monitored emission plumes from Northeast China, where ΔCO_2_ was larger in March–April 2022 than in the March–April 2015–2019 average. Our inversions suggest that the average anthropogenic CO_2_ emissions from the grid cells below the trajectories reaching RYO increased by 28% in March–April 2022 than in March–April 2019, in line with the observed increase of ΔCO_2_ (fig. S6F). The YON station captured emission plumes from coastal regions in North and East China. We observe a much larger reduction in ΔCO_2_ during January–March 2022 compared to the corresponding months in 2020 and 2021, which is consistent with the larger emission reductions over industrial and coastal provinces during the lockdown in 2022. During January–March 2022, the average CO_2_ emissions from the grid cells passed through by the corresponding backward trajectories were estimated to have declined by 11 to 46% in 2022 compared to those months in 2019. In January 2022, CO_2_ emissions over these areas declined by 26% compared to the 2019 level, corresponding to a negative ΔCO_2_ (fig. S6H), while CO_2_ emissions increased by 45% in January 2021 compared to the 2019 emissions, resulting in a positive ΔCO_2_ (fig. S6H). During April–May 2022, we lack enough air parcels coming from China to monitor CO_2_ emission changes.
