# Supplementary material for: Satellite reveals a steep decline in China’s CO2 emissions in early 2022
Source: Sci Adv. 2023 Jul 21;9(29):eadg7429. doi: 10.1126/sciadv.adg7429 (PMC10361590; doi:10.1126/sciadv.adg7429)
Supplement: Supplementary file 1 — Figs. S1 to S15 Tables S1 and S2 [file sciadv.adg7429_sm.pdf]

Science Advances
AAAS

Supplementary Materials for

Satellite reveals a steep decline in China's CO_2 emissions in early 2022

Hui Li *et al.*

Corresponding author: Bo Zheng, bozheng@sz.tsinghua.edu.cn

Sci. Adv. **9**, eadg7429 (2023)
DOI: 10.1126/sciadv.adg7429

This PDF file includes:

Figs. S1 to S15
Tables S1 and S2

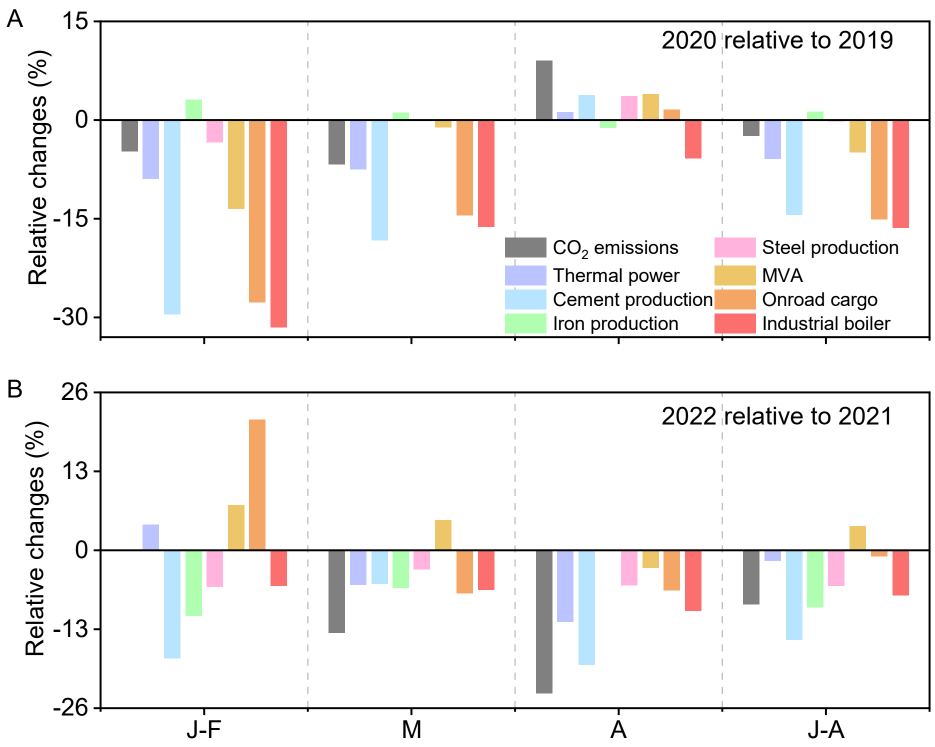

Fig. S1. Monthly relative changes in CO_2 emissions and industrial production from 2019 to 2020 (A) and from 2021 to 2022 (B). The industrial productions are derived from the National Bureau of Statistics (http://www.stats.gov.cn/).

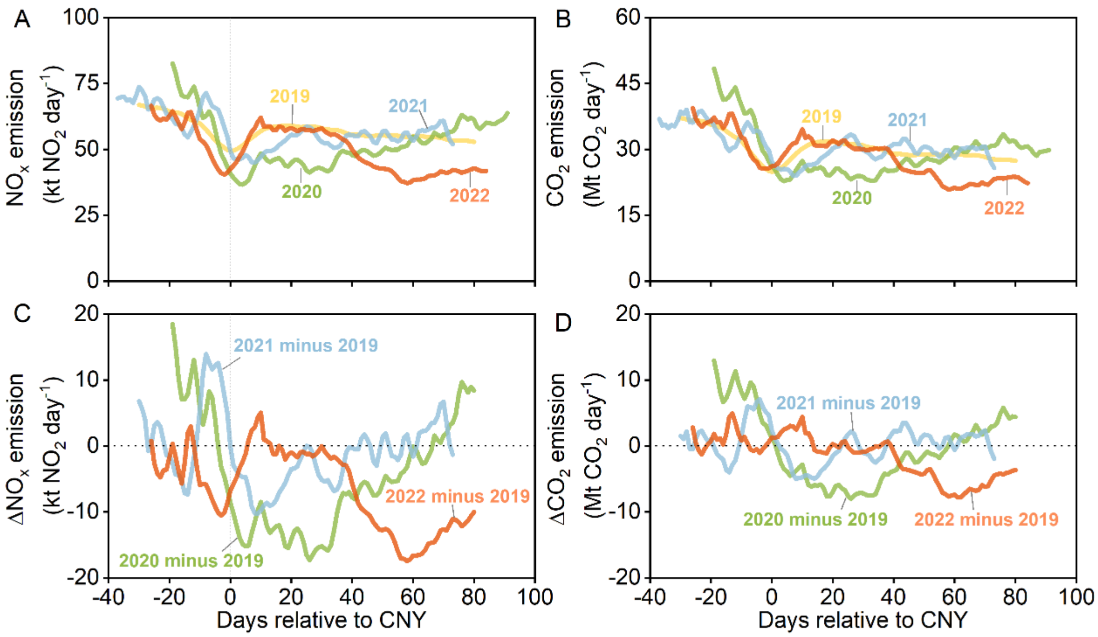

Fig. S2. Ten-day moving average NO_x and CO_2 emissions of China from January to April in 2019, 2020, 2021, and 2022. The 2019 emissions are derived from the MEIC emission inventory model, and the emission results from 2020 to 2022 are derived from the TROPOMI-constrained inversion estimates. (**A**) presents the 10-day moving average NO_x emissions and (**C**) presents the changes in NO_x emissions from 2019 to 2020, 2021, and 2022, respectively. (**B**) and (**D**) are plotted for CO_2 as (**A**) and (**C**). The days along x-axises represent the days relative to the Chinese New Year (CNY).

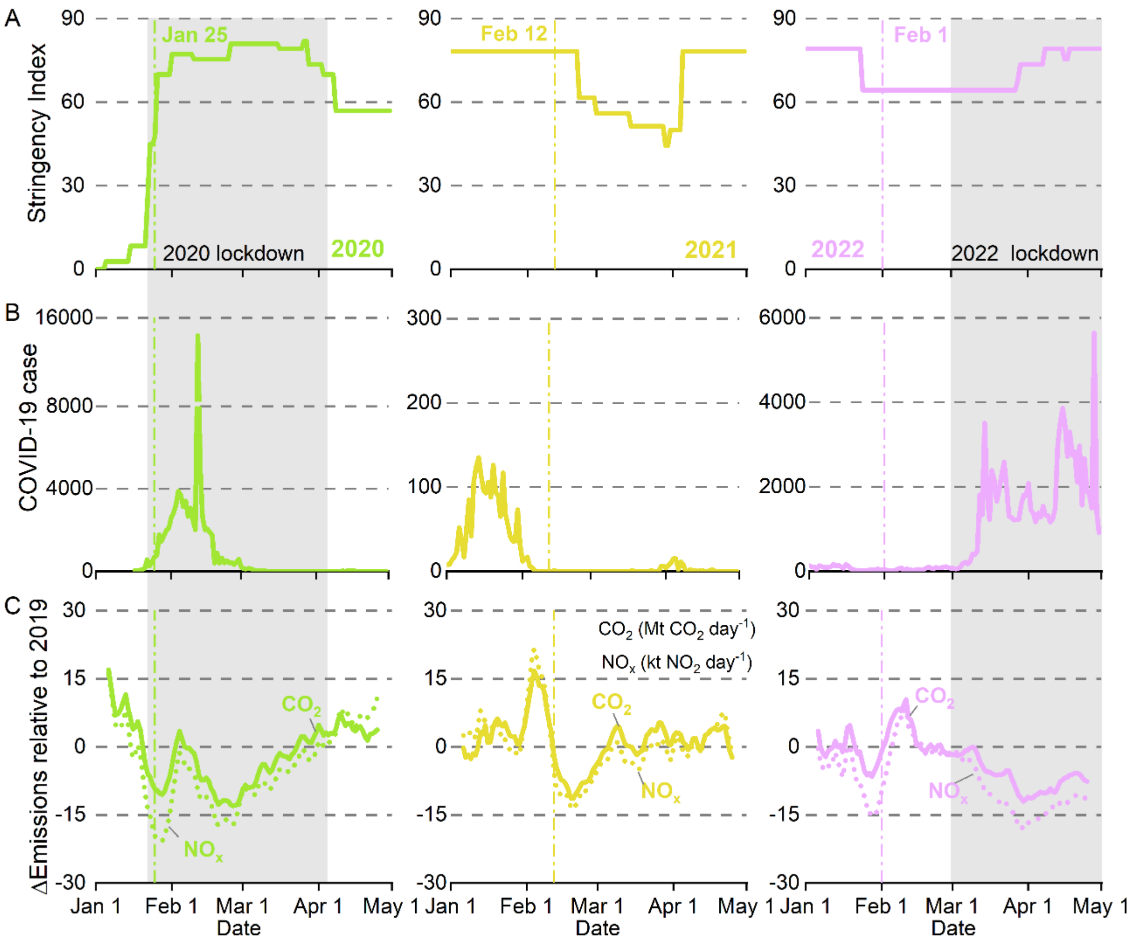

Fig. S3. Daily variation of COVID-19 policy stringency index, the daily confirmed cases, and the changes in CO_2 and NO_x emissions relative to 2019. (**A**) The daily stringency index (*36*), (**B**) daily new cases of COVID-19 reported in China (http://www.nhc.gov.cn/), and (**C**) the changes in China's emissions of CO_2 (solid curves) and NO_x (dashed curves) compared to the corresponding time in 2019. The vertical dashed lines represent the Chinese New Year. The gray shadings represent the lockdown periods in 2020 (23 January to 7 April) and 2022 (1 March to 30 April), respectively.

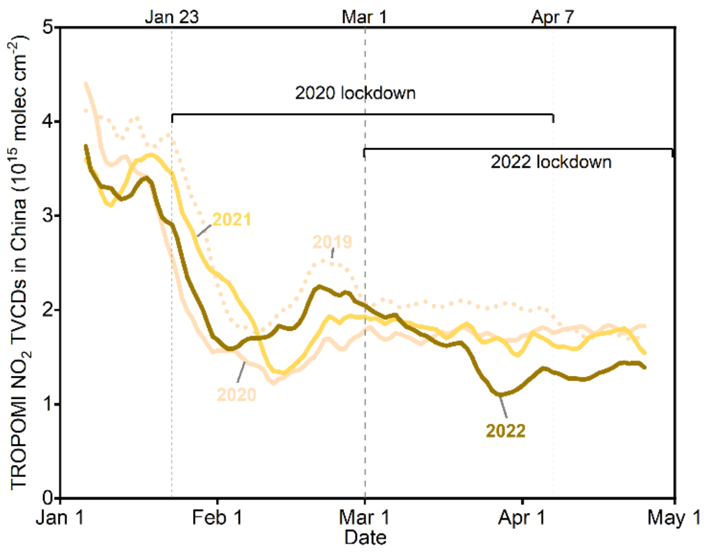

Fig. S4. Ten-day moving average TROPOMI NO_2 columns from January to April in 2019, 2020, 2021, and 2022. The grid cells dominated by anthropogenic sources (larger than 10^{15} molecules cm^{-2}) are used to estimate the national average levels. The curves for 2020, 2021, and 2022 represent the TROPOMI NO_2 columns excluding the influences of meteorological factors compared to 2019 based on the GEOS-Chem model simulations with the fixed emissions of 2019.

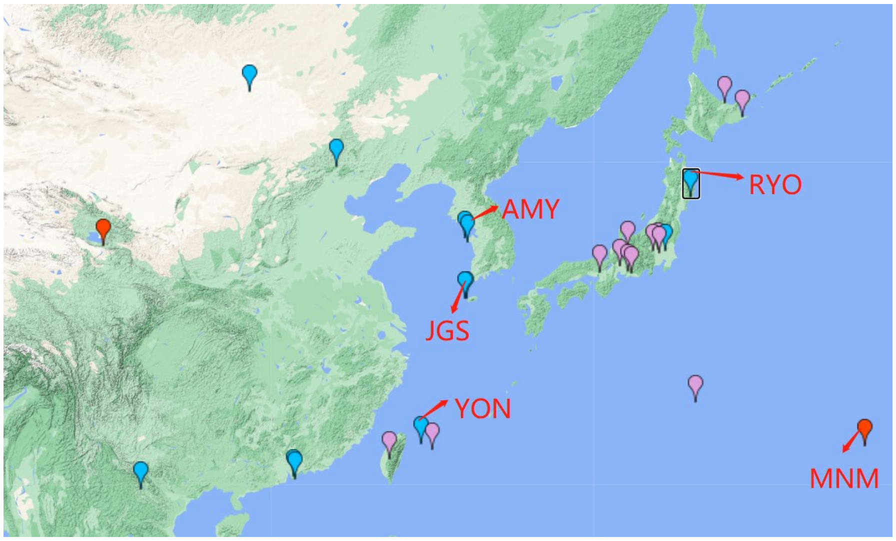

Fig. S5. The locations of AMY, JGS, RYO, YON, and MNM surface stations. Data are collected from World Data Centre for Greenhouse Gases (WDCGG, https://gaw.kishou.go.jp/)

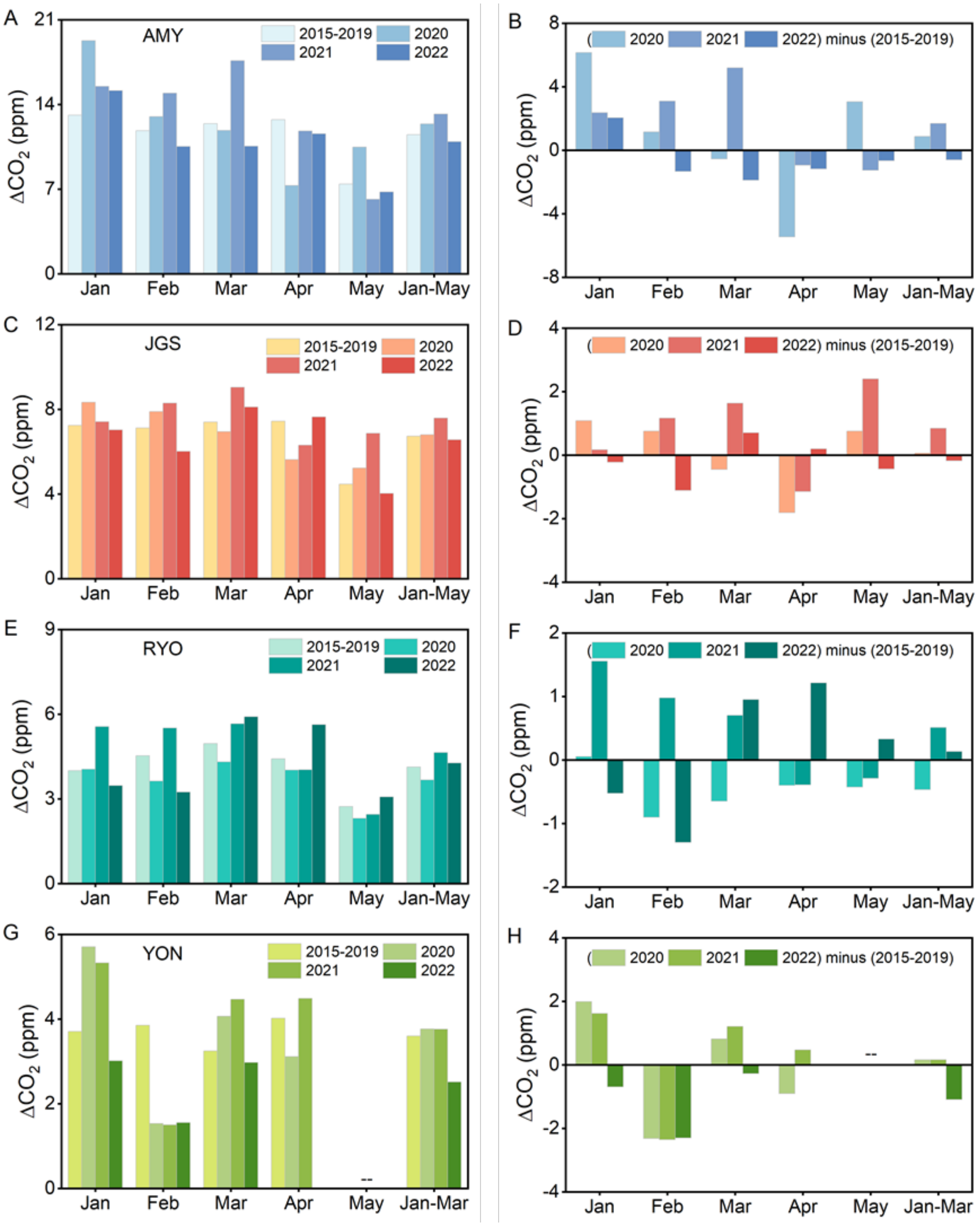

Fig. S6. The CO_2 enhancement (ΔCO_2) at four stations located downwind of China. CO_2 concentrations are collected from World Data Centre for Greenhouse Gases (WDCGG, https://gaw.kishou.go.jp/). (A) shows ΔCO_2 between January and May from 2015 to 2022, and (B) shows the changes of ΔCO_2 from the 2015-2019 mean to 2020, 2021, and 2022 based on the observation data from AMY. The same holds for JGS in (C) and (D), for RYO in (E) and (F), and for YON in (G) and (H). The short dashed lines in (G) and (H) represent a lack of enough valid data for May in YON (less than 1/3).

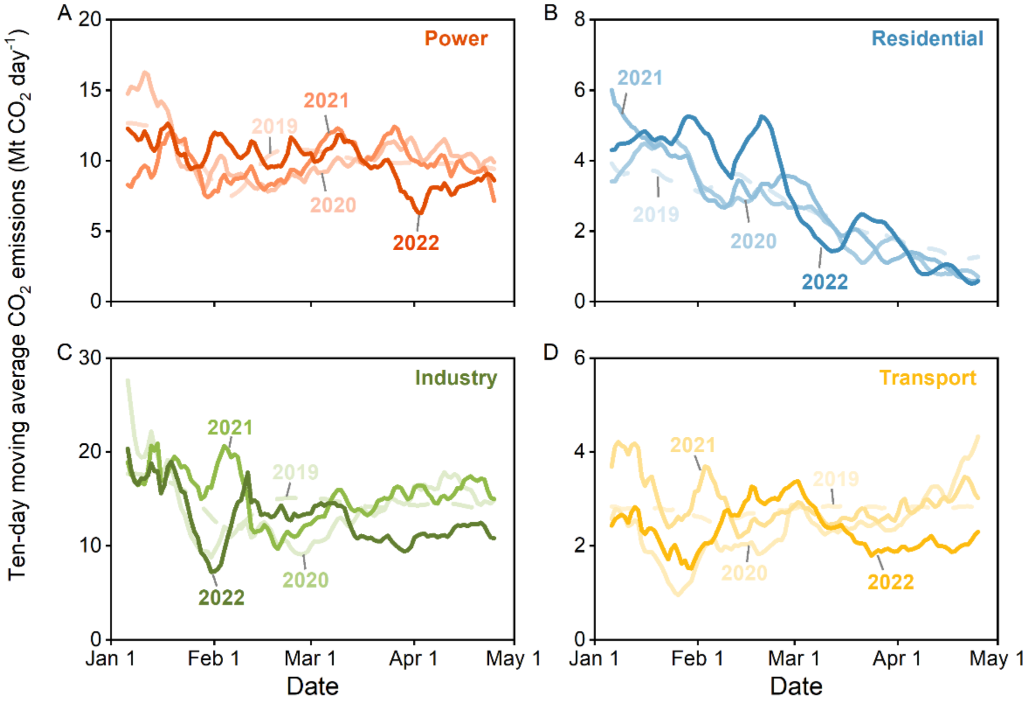

Fig. S7. Sector-specific ten-day moving average CO_2 emissions of China from January to April in 2019, 2020, 2021, and 2022.

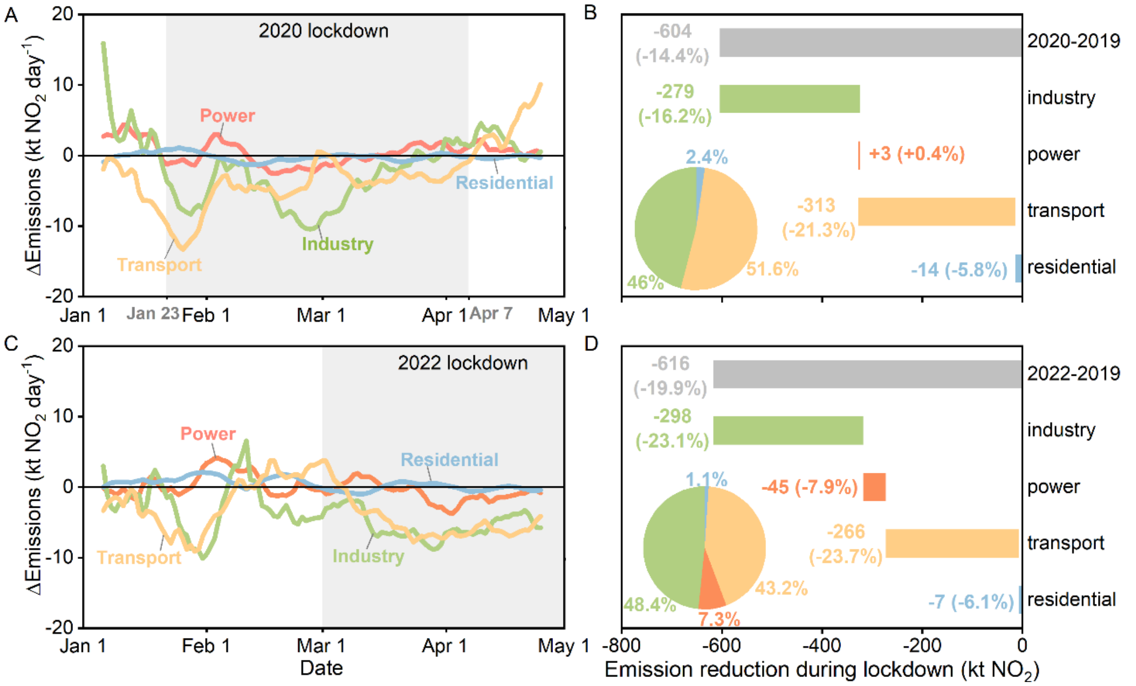

Fig. S8. Changes in the sectoral NO_x emissions of China in 2020 and 2022 compared to those in 2019. The ten-day moving average emission changes are shown for the power, industrial, transport, and residential sectors from 2019 to 2020 (**A**) and from 2019 to 2022 (**C**). The sector-specific NO_x emission reductions during the 2020 lockdown (gray shading in (**A**)) and the 2022 lockdown periods (gray shading in (**C**)) are presented in (**B**) and (**D**), respectively. The numbers in (**B**) and (**D**) show the absolute emission reductions and relative changes in emissions (in brackets) for each sector. The pie charts show the contribution of each sector to the total emission reductions during the lockdown periods.

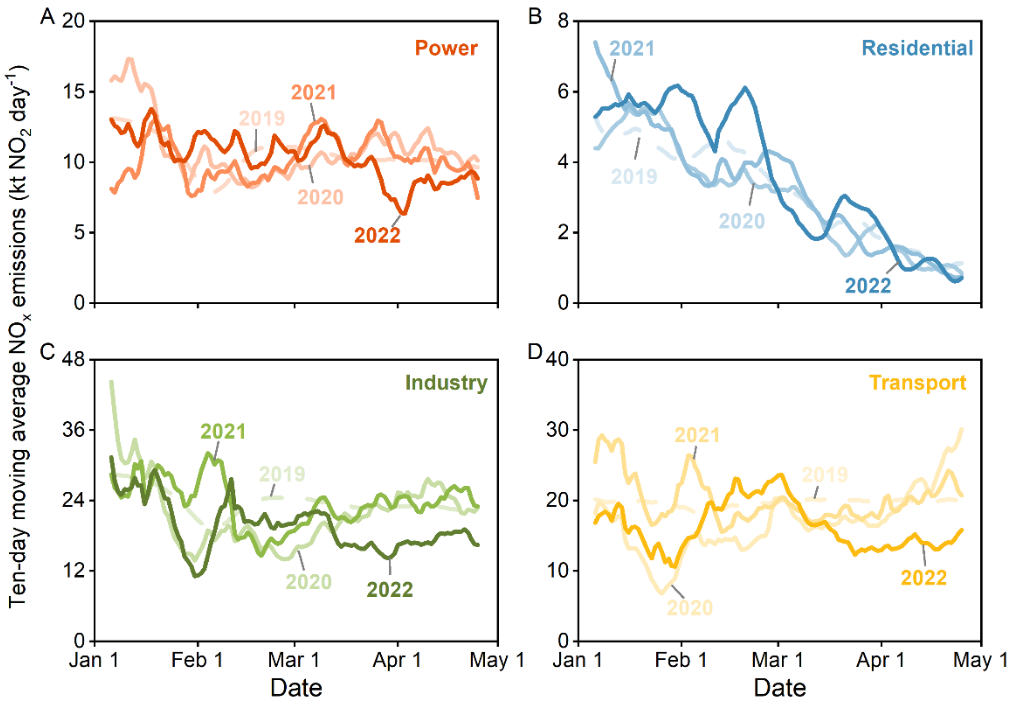

Fig. S9. Sector-specific ten-day moving average NO_x emissions of China from January to April in 2019, 2020, 2021, and 2022.

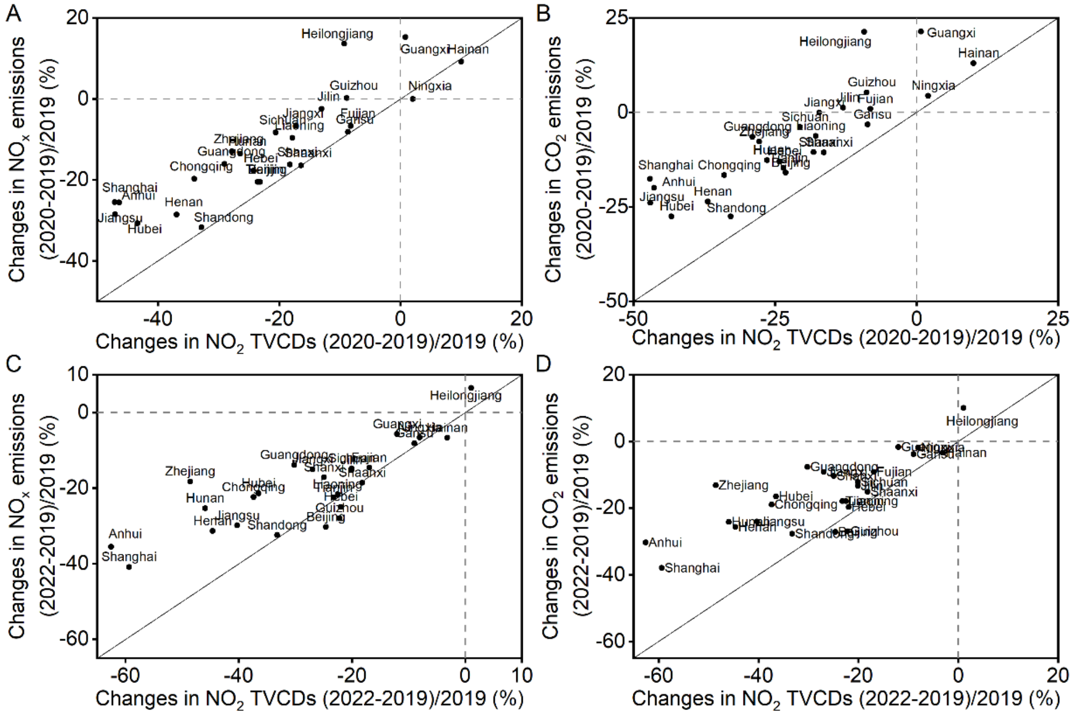

Fig. S10. Correlation between changes in TROPOMI NO_2 columns and changes in NO_x and CO_2 emissions by province from 2019 to 2020 and 2022. Each dot represents a province, which is plotted according to the changes in TROPOMI NO_2 columns along x-axises and changes in inversion-based emissions along y-axises. We only plot the provinces where TROPOMI NO_2 tropospheric vertical column densities larger than 1×10^{15} molecules cm^{-2} can cover more than 80% of their anthropogenic NO_x emissions. The influences of interannual variations of meteorological factors on NO_2 columns have been excluded through the GEOS-Chem model simulations with the fixed emissions of 2019.

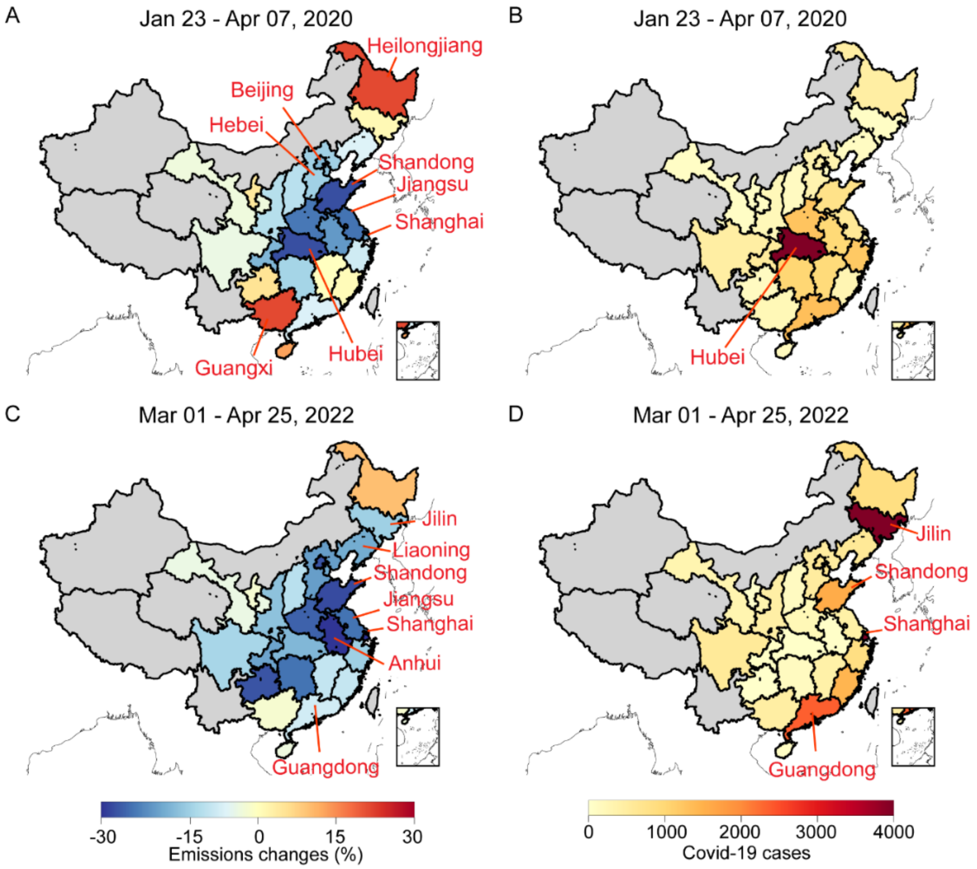

Fig. S11. Changes in provincial CO_2 emissions and COVID-19 cases in China during the 2020 and 2022 lockdowns. (**A**) and (**C**) represent the changes in provincial CO_2 emissions during the 2020 and 2022 lockdowns, respectively, compared to the corresponding periods in 2019. (**B**) and (**D**) represent the total number of COVID-19 cases during the lockdown in 2020 and 2022, respectively. We only plot the provinces where TROPOMI NO_2 tropospheric vertical column densities larger than 1×10^{15} molecules cm^{-2} can cover more than 80% of their anthropogenic NO_x emissions, while the provinces without adequate TROPOMI NO_2 observations are plotted in gray.

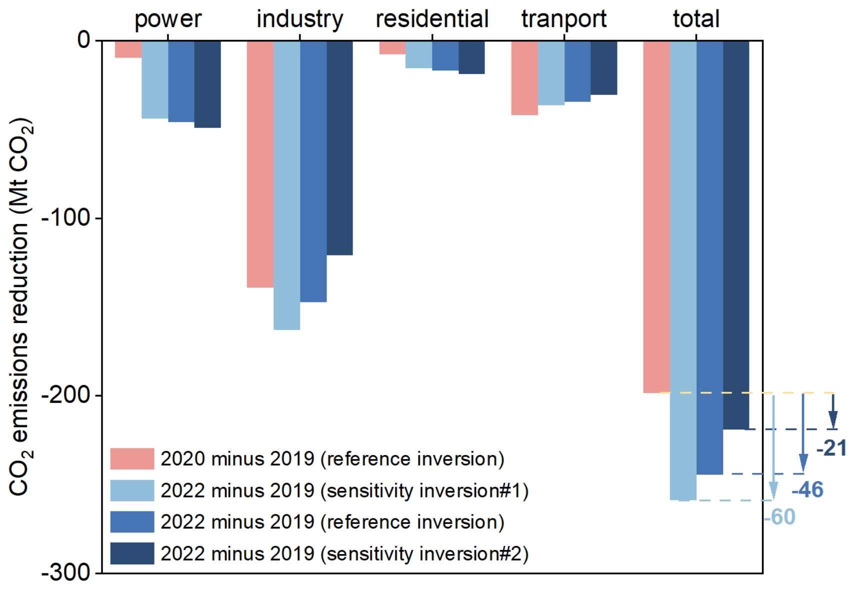

Fig. S12. The estimation of CO_2 emission reductions during lockdown in 2020 and 2022 based on different NO_x emission factors. The trends of NO_x emission factors used in different estimations are described in Table S2.

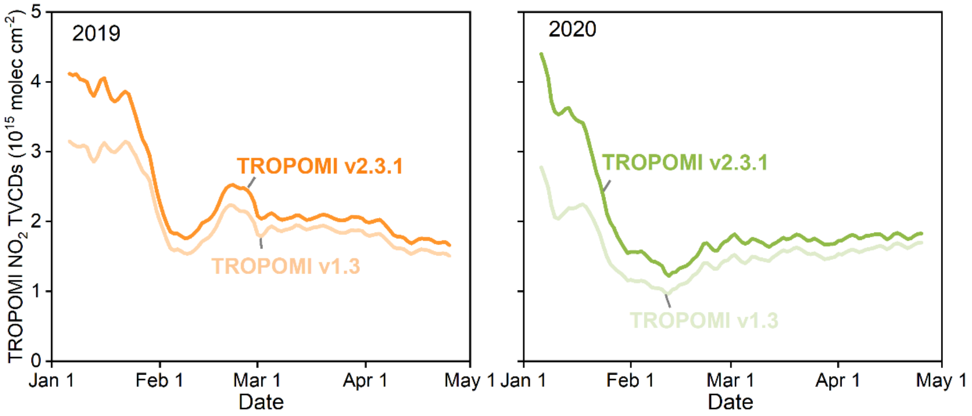

Fig. S13. Comparison of ten-day moving average NO_2 columns between the TROPOMI versions 1.3 and 2.3.1.

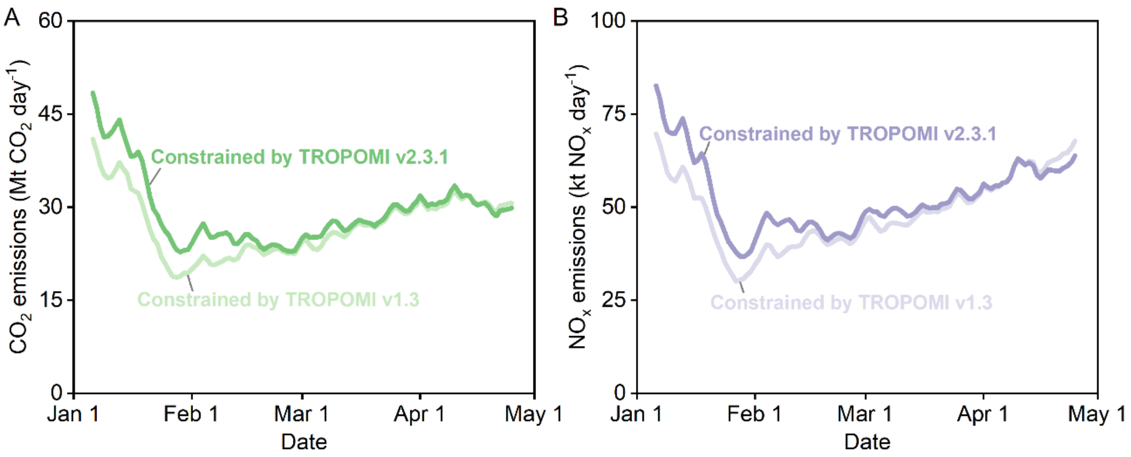

Fig. S14. Comparison of inversion-estimated emissions in 2020 constrained by the TROPOMI NO_2 columns of versions 1.3 and 2.3.1.

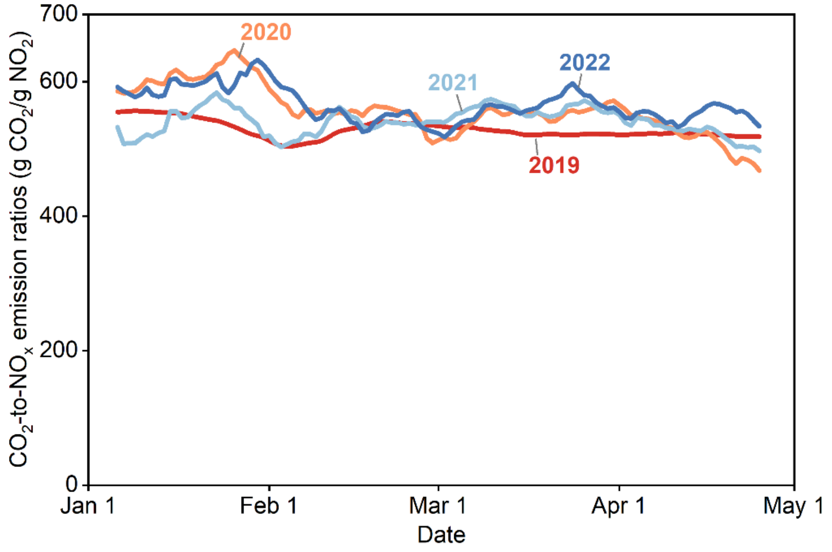

Fig. S15. Ten-day moving average CO_2-to-NO_x emission ratios for 2019, 2020, 2021, and 2022 in this study.

Table S1. Comparison of inversion-estimated emissions in 2020 constrained by the TROPOMI NO_2 column of version 1.3 and version 2.3.1.

			NO_x emissions (Mt NO_2)		
**Month**	**2019**	**2020_v1.3**	**2020_v2.3.1**	**Diff_v1.3 (%)**	**Diff_v2.3.1 (%)**
Jan	1.6	1.2	1.5	−23.7	−8.2
Feb	1.5	1.2	1.3	−24.6	−16.7
Mar	1.7	1.5	1.6	−14.2	−10.7
Apr	1.4	1.5	1.5	11.6	9.5
Jan-Apr	6.3	5.4	5.8	−13.7	−7.2
			**CO_2 emissions (Mt CO_2)**		
**Month**	**2019**	**2020_v1.3**	**2020_v2.3.1**	**Diff_v1.3 (%)**	**Diff_v2.3.1 (%)**
Jan	887	757	904	−14.7	2.0
Feb	813	645	714	−20.6	−12.1
Mar	919	830	856	−9.6	−6.8
Apr	706	768	770	8.7	9.0
Jan-Apr	3324	3000	3245	−9.7	−2.4

Table S2. The ratios of sectoral NO_x emission factor relative to 2019 used in our inversions.

	Description	Sectors	2020	2021	2022
Reference inversion	The percentage decrease in NO_x emission factor is half of that during the last year.	Cement	0.93	0.90	0.88
		Iron	0.90	0.86	0.83
		On-road	0.98	0.97	0.97
Sensitivity inversion #1	Unchanged Since 2020	Cement	0.93	0.93	0.93
		Iron	0.90	0.90	0.90
		On-road	0.98	0.98	0.98
Sensitivity inversion #2	The exponential decrease in NO_x emission factors.	Cement	0.93	0.86	0.80
		Iron	0.90	0.81	0.73
		On-road	0.98	0.96	0.94
